# Crew-Friendly Countermeasures Against Musculoskeletal Injuries in Aviation and Spaceflight

**DOI:** 10.3389/fphys.2020.00837

**Published:** 2020-07-10

**Authors:** Daniel K. O’Conor, Sawan Dalal, Vignesh Ramachandran, Bethany Shivers, Barry S. Shender, Jeffrey A. Jones

**Affiliations:** ^1^Center for Space Medicine, Baylor College of Medicine, Houston, TX, United States; ^2^Human Systems Engineering, Naval Air Warfare Aircraft Division, Patuxent River, MD, United States; ^3^Commander Fleet Logistics Support Wing, Commander Naval Air Force Reserve, United States Navy Reserves, Naval Air Station JRB Fort Worth, TX, United States

**Keywords:** musculoskeletal injuries, spaceflight, aviation, countermeasures, resistive exercise

## Abstract

Aviation and space medicine face many common musculoskeletal challenges that manifest in crew of rotary-wing aircraft (RWA), high-performance jet aircraft (HPJA), and spacecraft. Furthermore, many astronauts are former pilots of RWA or HPJA. Flight crew are exposed to recurrent musculoskeletal risk relating to the extreme environments in which they operate, including high-gravitational force equivalents (g-forces), altered gravitational vectors, vibratory loading, and interaction with equipment. Several countermeasures have been implemented or are currently under development to reduce the magnitude and frequency of these injuries. Cervical and lumbar spine, as well as extremity injuries, are common to aviators and astronauts, and occur in training and operational environments. Stress on the spinal column secondary to gravitational loading and unloading, ± vibration are implicated in the development of pain syndromes and intervertebral disk pathology. While necessary for operation in extreme environments, crew-support equipment can contribute to musculoskeletal strain or trauma. Crew-focused injury prevention measures such as stretching, exercise, and conditioning programs have demonstrated the potential to prevent pre-flight, in-flight, and post-flight injuries. Equipment countermeasures, especially those addressing helmet mass and center of gravity and spacesuit ergonomics, are also key in injury prevention. Furthermore, behavioral and training interventions are required to ensure that crew are prepared to safely operate when faced with these exposures. The common operational exposures and risk factors between RWA and HPJA pilots and astronauts lend themselves to collaborative studies to develop and improve countermeasures. Countermeasures require time and resources, and careful consideration is warranted to ensure that crew have access to equipment and expertise necessary to implement them. Further investigation is required to demonstrate long-term success of these interventions and inform flight surgeon decision-making about individualized treatment. Lessons learned from each population must be applied to the others to mitigate adverse effects on crew health and well-being and mission readiness.

## Introduction

The crew of high-performance jet aircraft (HPJA), rotary-wing aircraft (RWA), and spacecraft are subjected to extreme occupational conditions that elevate their risk of pre-flight, in-flight, and post-flight musculoskeletal pathology and disability. In astronauts, musculoskeletal issues are the second most common cause of in-flight complaints (second only to sleep disturbance) with an incidence of 3.34 events/person-year (Scheuring, 2010). Musculoskeletal issues in HPJA and RWA pilots are similarly ubiquitous with a 1-year prevalence of neck pain in HJPA pilots as high as 83% and back pain in RWA pilots as high as 90% (Lange et al., 2011; Murray et al., 2017). The same period prevalence for neck and back pain in the general population is approximately 37 and 15%, respectively (Andersson, 1999; Fejer et al., 2006). The musculoskeletal issues in crew manifest as pain, impaired concentration and situational awareness, impaired motor control and posture, inability to perform in-flight maneuvers, grounding, lost work or training time, increased utilization of medical resources, and forced retirement (Kikukawa et al., 1995; Phillips, 2011; Shiri et al., 2015; Posch et al., 2019). Evidence suggests that many pilots do not report pain or injury and continue to fly because of fear of losing flight status (Kikukawa et al., 1995; Kerstman et al., 2012; Posch et al., 2019).

The risks are compounded in some astronauts who were former HPJA or RWA pilots and later participate in spaceflight. In one study of intervertebral disk (IVD) injury in a group of 321 astronauts, over half (178) of the astronauts were former HPJA pilots (Johnston et al., 2010). Moreover, all astronauts train in the T-38 aircraft, a super-sonic jet trainer. While the T-38 is not considered a HPJA, it is capable of acrobatic maneuvers and musculoskeletal pain and injury have been reported in pilots of this type of aircraft (Wagstaff et al., 2012).

**FIGURE 1:**
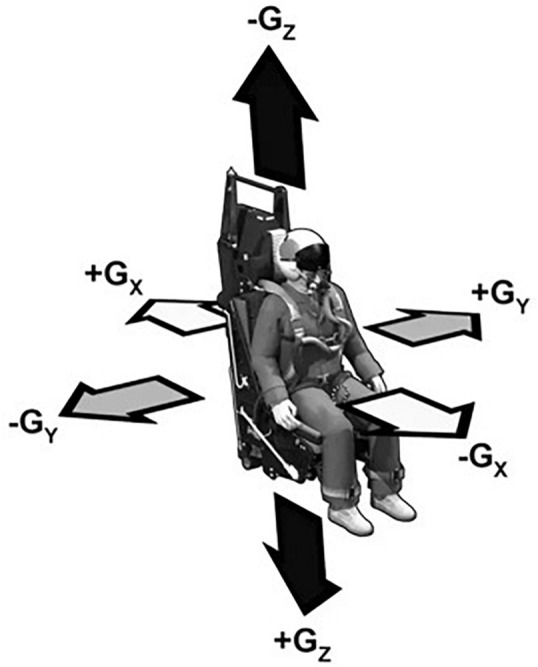
Gravitational (Gx, Gy, and Gz) axes with respect to aircraft pilot or astronaut. Retrieved from https://www.quora.com/Are-astronauts-and-fighter-pilots-trained-for-only-positive-Gs. Original source from Air Force training materials.

**FIGURE 2:**
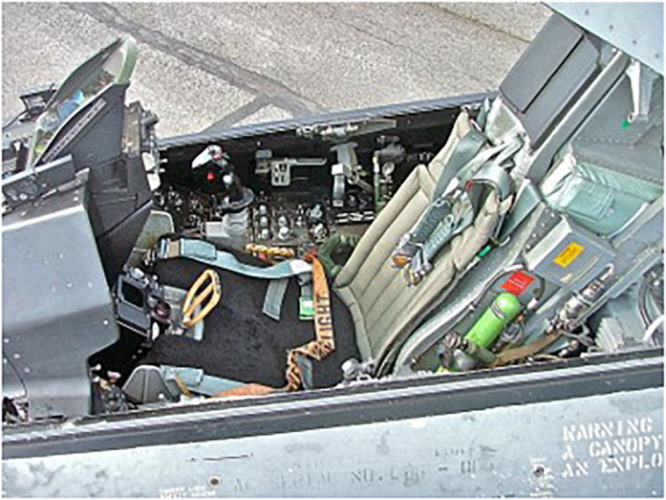
“Cockpit of F-16,” by Edvard Majakari is licensed under “CC BY 3.0.” HPJA showing configuration of seatback angle and controls in relation to pilot seat.

Musculoskeletal pain and injury in these populations have been attributed to several aspects of flight including: microgravity; high-g-forces acting parallel to axis of the spine (high-Gz, Figure 1); vibratory loading, eccentrically loaded head-supported mass (HSM); movements required for environmental awareness; cockpit layout or equipment configuration (Figure 2); impact associated with ejection or hard-landing; fatigue and overuse; and physical training (Jones et al., 2000). While microgravity is an exposure exclusive to spaceflight, all other exposures are experienced to some degree by both populations.

To counter the debilitating effects of the occupational injuries associated with the aerospace environment, many efforts have been made to develop both aircraft- and pilot-focused countermeasures. Many of the countermeasures developed out of needs identified in HPJA crew, but application to astronauts and crew of other aircraft should be considered due to their similarly extreme exposure. This study aims to review the countermeasures available to aerospace medicine professionals in support of their mission to protect crew from the conditions that might otherwise predispose them to injury and disease. Some potential countermeasures include exercise, stretching, traction, behavioral interventions, re-design of cockpit or equipment, helmet re-configuration and counterweights, and anti-vibration seating (Table 1).

**TABLE 1:** Exposures, mechanism of injury, effects, and countermeasures.

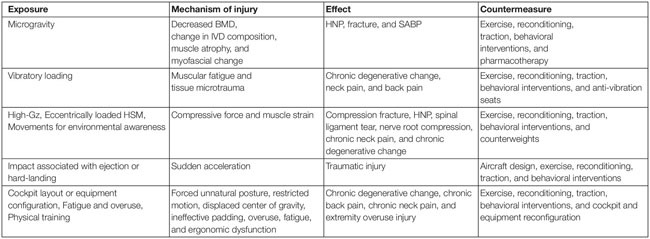

## Microgravity

In order to develop effective countermeasures, it is critical to understand the underlying mechanism of injury. Loss of gravitational loading in spaceflight results in decreased bone mineral density (BMD), change in IVD composition, flattening of the spinal curvature, muscle atrophy, and other myofascial changes in astronauts (Malko et al., 1999; Andreoni et al., 2000; LeBlanc et al., 2000a, b; Chang et al., 2016; Bailey et al., 2018). BMD loss disproportionately affects weight-bearing areas of the body including the spine, pelvis, and legs while other areas of the body such as the arms may be relatively spared (LeBlanc et al., 2000b; Vico and Hargens, 2018). Furthermore, BMD loss does not uniformly affect cortical and trabecular bone (Vico et al., 2000). Cortical thinning has been observed in load-bearing bone and the greatest loss of bone mass occurs in the cortical bone while trabecular bone undergoes a larger percentage loss than cortical bone (Lang et al., 2004). Computer modeling suggest these changes in both cortical and trabecular bone result in significant overall loss of bone strength that predisposes to injury and long-term sequelae if reconditioning does not sufficiently recover pre-flight strength (Keyak et al., 2009).

Although studies of astronauts immediately post-flight detected no appreciable change in IVD height, analog studies suggest unloading of the spine allows increased water absorption by proteoglycans of the IVD, leading to an increase in IVD volume (Malko et al., 1999; Chang et al., 2016). IVD expansion can further contribute to annulus fibrosis weakening by impairing the avascular, diffusion-limited nutrition of the disk (Urban et al., 2004; Johnston et al., 2010). Studies in rat analogs flown in space showed decreased collagen in the IVD that may weaken its structure (Foldes et al., 1996). This suggests a degenerative component analogous to that implicated in the development of spine injury in other aviators, especially RWA pilots subjected to whole body vibration (WBV) (Walters et al., 2013). Expansion of the anterior and posterior spinal ligaments in microgravity may contribute to risk by destabilizing the spine (Johnston et al., 2010). Finally, muscle atrophy and a change in relative abundance of Type I muscle fibers results in deep spinal muscles that are more prone to isometric fatigue (Scheuring, 2010; Sayson et al., 2015).

**FIGURE 3:**
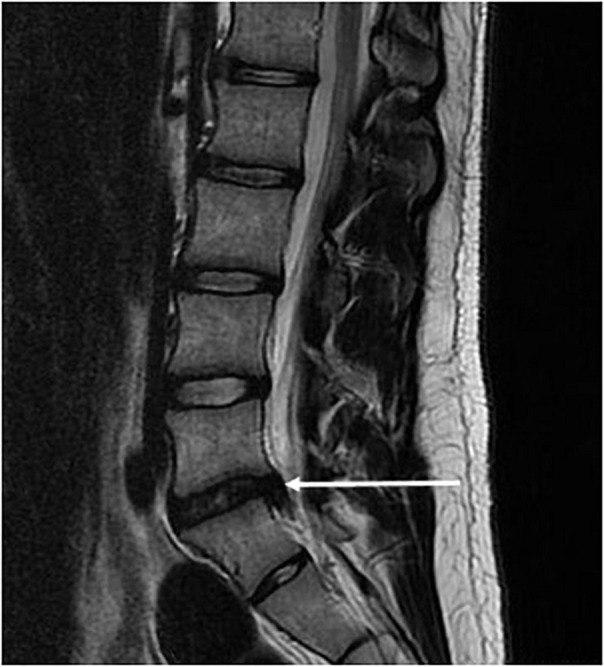
MRI of lumbar spine showing HNP, arrow added to original figure.

**FIGURE 4:**
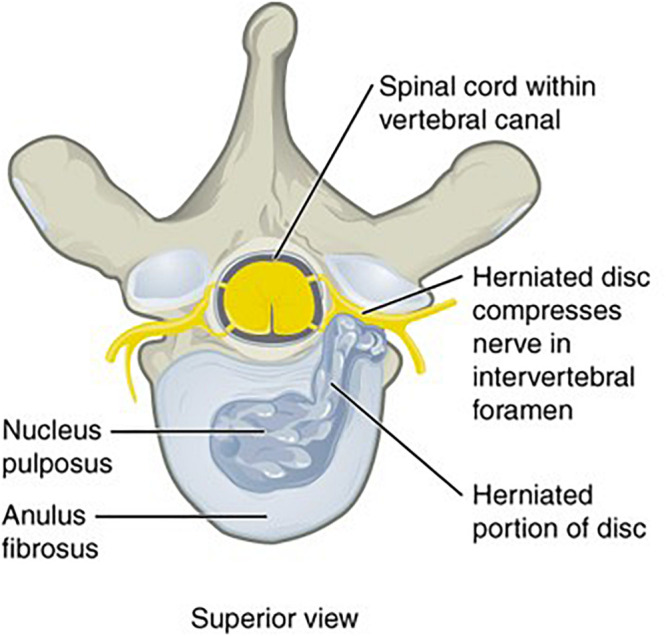
“Herniated Disk,” by OpenStax College is licensed under “CC BY 3.0.” Vertebral body and IVD of human spine, showing HNP. Original figure cropped to remove accompanying radiographic imaging.

In microgravity, the vertebral body BMD changes and loss of paraspinal lean muscle mass predispose to herniated nucleus pulposus (HNP) (Figures 3, 4) (Johnston et al., 2010; Chang et al., 2016). The risk of HNP is greatest in the first-year post-flight secondary to the high Gz and impact-loading upon return to Earth and re-acclimation to Earth’s gravity (Johnston et al., 2010). HNP can be serious; one astronaut experienced immediate post-flight HNP requiring hospitalization and cervical spine discectomy (Johnston et al., 2010). While astronauts with experience as pilots of HPJA were not shown to have increased post-spaceflight risk of HNP relative to other astronauts, all astronauts could be considered subjects of high Gz-loading as part of T-38 flight training and the conditions of launch and landing (Johnston et al., 2010).

Bone mineral density loss can also predispose to fracture, but none of over 50 fractures in active United States astronauts can be definitely attributed to exposure to microgravity (Ramachandran et al., 2018a). Two astronauts experienced fractures following long-duration (∼6 months) missions on the International Space Station (ISS), one a fracture of the fibula following a slip and fall on ice, and the other a femoral neck fracture from a jump of 2.5 feet (Scheuring, 2016). The concern remains that loss of BMD and changes in bone architecture in microgravity can contribute to the risk of injury in astronauts which may persist for months after return to Earth’s gravity (Lang et al., 2004; Vico et al., 2017).

In the astronaut population, HNP incidence is not increased with longer duration missions on the ISS, likely due to protocols that limit vertical positioning and ambulation within the first 24 h post-flight and reconditioning coaches to facilitate protected recovery for the first 2 weeks post-flight (Johnston et al., 2010). The ability of pre-flight strengthening to reduce the risk of HNP in astronauts has been speculated but not established (Johnston et al., 2010). In-flight countermeasures are difficult to employ because only sustained axial loading would likely decrease the disk volume enough to counteract the changes seen in microgravity, and exercise countermeasures are limited to 2 h/day (Johnston et al., 2010). Any proposed countermeasures would have to be configured to fit within the confines of the spacecraft and not interfere with mission objectives. On the other hand, anecdotal reports from astronauts suggest that intermittent loading could have some benefit (Sayson et al., 2015). The use of the penguin compression suit by Russian cosmonauts may be related to the decreased incidence of back pain in cosmonauts (Sayson et al., 2015). Furthermore, astronauts report that the shoulder pressure they experience in the extravehicular mobility unit (EMU) during EVA decreases with subsequent EVAs, possibly as a result of the sustained effect of intermittent loading imparted by the suit (Sayson et al., 2015).

Countermeasures to preserve muscular strength and endurance and BMD in astronauts include aerobic and resistive exercise together with nutritional supplementation, high dose vitamin D, and bisphosphonates (LeBlanc et al., 2013; Scheuring, 2016; Swaffield et al., 2018). A study of astronauts aboard the ISS, has shown that the bisphosphonate therapy together with resistive exercise is more effective in preserving BMD than exercise alone (Sibonga et al., 2019). Researchers have proposed that the current exercise devices available on the ISS could be augmented by a constant-resistance pulley to prevent atrophy of spinal muscle stabilizers (Sayson et al., 2015). A possible exercise regimen on this device would employ low resistance and high repetition to build Type I muscle fibers necessary for postural stability (Sayson et al., 2015). A pulley exercise device has been proposed as the most effective form of resistance because elastic bands are known to provide increasing resistance throughout the repetition and would be less appropriate for building Type I fibers (Sayson et al., 2015).

Another consequence of microgravity is space adaptation back pain (SABP). SABP is a self-limited condition involving pain predominantly in the lumbar spine during the first several days of spaceflight (Kerstman et al., 2012). Increased IVD hydration in the microgravity environment stretches annular collagen causing activation of mechanoreceptors and free nerve endings that communicate a perception of pain (Sayson et al., 2013). For SABP, stretching the lumbar spine or assuming a fetal tuck position are the most efficacious countermeasures with at least 90% of astronauts reporting symptom relief when performing these maneuvers (Kerstman et al., 2012). This fetal tuck position activates the spinal flexors which reintroduces a compressive load to the IVDs while simultaneously opening the intervertebral foramen to alleviate pressure on the spinal nerve roots (Sayson et al., 2013, 2015). Exercising on the cycle ergometer or the treadmill with vibration and isolation system (TVIS) were associated with symptom relief in 85% of astronauts, either as a result of the exercise motion itself or the compressive loading of the TVIS harness (Kerstman et al., 2012).

The goals of countermeasures in spaceflight include minimizing adverse health outcomes and lifetime health risks, facilitating in-flight performance, and optimizing post-flight recovery (Scheuring, 2016). Injuries in-flight could compromise the ability to conduct EVA, and land or egress a spacecraft (Scheuring, 2016). Specialized post-flight reconditioning includes flexibility training, cardiovascular conditioning, resistive exercise, and massage (Scheuring, 2016). Post-flight rehabilitation includes limitations on spinal flexion which is considered a risk factor for post-flight HNP (Belavy et al., 2016).

## Whole Body Vibration

Whole body vibration is a risk most prominent in RWA crew but also present in HPJA crew and astronauts (Pippig, 1994; Jones et al., 2015). WBV results in musculoskeletal injury through muscular fatigue, tissue microtrauma, and chronic degenerative change (Walters et al., 2013). The vibrations experienced by the aviator vary based on posture and mode of flight (velocity, acceleration, and maneuvers being conducted) and how the vibrations are amplified by the seat, airframe, or controls (Walters et al., 2013; Baig et al., 2014). WBV is a contributing factor to neck pain in pilots and chronic degenerative changes of the spine (Walters et al., 2013). Some of these issues have been addressed in the RWA community with the introduction of anti-vibration seats (Phillips, 2011). A study of one such anti-vibration intervention supports its efficacy in reducing strain on crew neck muscles (Wright Beatty et al., 2018).

## High-Gz, Eccentrically Loaded HSM, Movements for Environmental Awareness

High-Gz exposure has been established as a risk factor for neck pain based on the relative increased incidence of neck pain in pilots of high-Gz aircraft compared to pilots of intermediate- and low-Gz aircraft (Jones et al., 2000). High-Gz loading has been known to cause compression fracture, HNP, and spinal ligament tear (Shiri et al., 2015). Compressive force on the bony vertebral lamina causing nerve root injury is the proposed mechanism for pain experienced in-flight (Jones et al., 2000).

Evidence for compression of IVD during flight is supported by decreased body height following aerial combat maneuvers (Johnston et al., 2010). Chronic pain can result from nerve injury as well as HNP secondary to degenerative changes and long-term effects of compressive forces of flight (Johnston et al., 2010). The weight of the helmet worn by pilots together with night vision goggles (NVGs) both increases the weight supported by the cervical vertebrae and neck musculature and alters the center of gravity of the head. An off-center, heavier-than-physiologic HSM under high-G loading places considerable strain on the pilot’s neck.

High-Gz loading in conjunction with HSM is further exacerbated with several head positions and movements, most notably the “check six,” a combat maneuver in which the pilot maximally rotates the head to look behind the aircraft (Kerstman et al., 2012; Wagstaff et al., 2012; Shiri et al., 2015). Furthermore, wearing HSM like NVGs (and battery pack) for long periods of time during certain conditions of flight such as air-to-air combat maneuvers have been reported to require excessive neck muscle activation, contributing to exacerbation of in-flight pain (Äng and Kristoffersson, 2013). Cumulative time wearing NVGs has been implicated as an independent risk factor in the development of neck pain with 90% of helicopter pilots experiencing neck pain once they had logged greater than 150 h wearing NVGs (Posch et al., 2019). NVGs also restrict the field of vision, requiring the pilot to make more exaggerated head movements to maintain situational awareness and thereby increasing stress on the neck (Thoolen and van den Oord, 2015). One component of safety equipment, the horse collar (water flotation unit), has been known to exert pressure on the neck and force the head forward in flight (Thoolen and van den Oord, 2015). In the event of an impact or ejection, additional HSM could increase the risk of a neck injury (Parr et al., 2013).

Efforts have been made to mitigate these environmental effects with helmet counterweights, anti-vibration seats, and re-configuration of cockpits and equipment (Harrison et al., 2007; Wright Beatty et al., 2018). The need for pilots to have rapid, hands-free access to communication and navigational information has fueled the development of head-mounted electronic equipment. These avionics demand placement of an eccentrically mounted mass which increases neck strain even as newer helmets are being designed with lightweight materials. Counterweights may be employed to maintain physiologic center of gravity but additional mass contributes to the total load applied to the cervical vertebrae. Regardless, counterweight use has been shown to reduce muscle activation in pilot’s wearing NVGs (Harrison et al., 2007). An alternative to a counterweight is a spring-loaded mechanism designed to lower the inertia of the head while wearing a HSM (Smith, 2016). Lighter helmet configurations are protective against neck pain and advancement in material technology could further mitigate this factor (Johnston et al., 2010). In the development of future helmets and head-mounted equipment, it is imperative that the mass as well as the center of gravity be considered to minimize the risk of head and neck injury (Shender et al., 2001). The masses that must be considered include the helmet, visor, oxygen mask, hoses, communication cables, additional displays, and chemical-biological hoods (Shender et al., 2001).

For HPJA crew, efforts to consider head position before initiating high-G maneuvers have shown to be protective against neck pain (Johnston et al., 2010). In one study of F/A 18 pilots, 69% reported employing pre-positioning maneuvers of the head or bracing of the head against cockpit structures prior to initiating a high G maneuver in order to prevent pain (Drew, 1999). While doing so may limit performance, pilots of HPJA reported that refraining from movement or only moving the head in one axis at a time above a certain G threshold helped them to mitigate pain (Wagstaff et al., 2012).

## Exercise Countermeasures

Numerous countermeasures have been developed to mitigate the musculoskeletal effects on aircrew and astronauts including exercise, stretching, reconditioning, traction, and behavioral interventions (Hamalainen et al., 1998; Drew, 1999; Alricsson et al., 2004; Ang et al., 2009; Salmon et al., 2013; Chumbley et al., 2016; Scheuring, 2016; Ramachandran et al., 2017, 2018b, 2019, 2020; Dalal et al., 2019). Even in the absence of structured or directed countermeasures, surveys of pilots have indicated successful self-directed attempts at mitigating pain or injury (Jones et al., 2000). A self-reported survey of pilots who conducted self-directed stretching prior to flight indicated that such precautions were not associated with significant reduction in pain, but self-directed weight training has been reported as an effective measure with 62% of pilots in one study endorsing its efficacy (Kikukawa et al., 1995; Jones et al., 2000).

**FIGURE 5:**
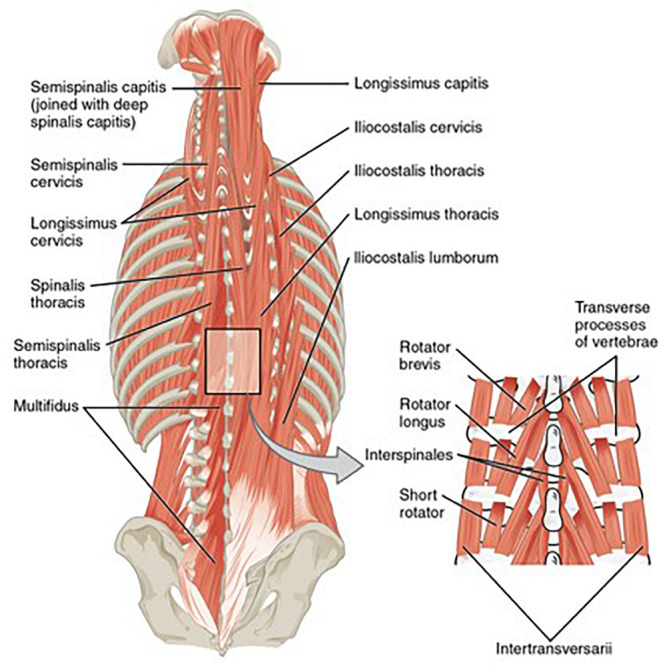
“Muscles of the Back” by OpenStax College is licensed under “CC BY 3.0.” Deep cervical muscles targeted by the resistive exercise regimen.

Much like the evidence in support of directed exercise interventions for non-specific neck pain in the general population, studies have also demonstrated success in RWA and HPJA pilots using a prescribed resistive exercise regimen with elastic bands to improve strength and endurance of cervical spine musculature (Figure 5), mitigate neck pain, and reduce time removed from flying duties due to injury (Hamalainen et al., 1998; Hurwitz et al., 2008; Ang et al., 2009; Bronfort et al., 2010; Salmon et al., 2013). Increased strength and endurance of neck muscles has observed both among RWA and HPJA crew who undertook training with bodyweight isometric exercise or resistive exercise (Alricsson et al., 2004; Salmon et al., 2013). HPJA pilots undertaking a year-long dynamic exercise regimen with hand weights and stretching were shown to require fewer restrictions of high Gz flight than pilots training with passive motion of the neck while wearing a weighted flight helmet, but both groups showed increased strength of the cervical spinal muscles (Hamalainen et al., 1998). Reduction of reported neck pain symptoms has been achieved through the use of supervised physiotherapy with non-postural, postural, and elastic band-resisted exercise among RWA crew (Ang et al., 2009).

**FIGURE 6:**
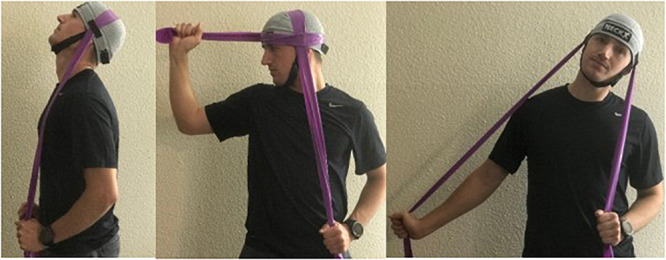
Portable cervical exercise device exercises in three axes of movement (rotation, flexion/extension, and lateral bending).

With a focus on implementing portable countermeasures, a wearable cervical spine resistive exercise device (Figure 6) has been shown to be effective in improving cervical spine strength and endurance over the course of a 6-week intervention (Ramachandran et al., 2017, 2018b, 2019, 2020; Dalal et al., 2019). Pilots with neck pain have reduced cervical spine range of motion compared to healthy pilots and efforts have been made to study the effect of exercise interventions on cervical spine range of motion (De Loose et al., 2009). Subjects undertaking exercise with a portable cervical spine resistive exercise device (PCED) demonstrated increased cervical spine range of motion in addition to decreased need for anti-inflammatory medication and decreased frequency of reported neck pain. In this study, mean strength among participants increased: flexion (+50%), extension (+38%), lateral bend (+35%), and rotation (+28%). Mean endurance also increased: flexion (+70%) and extension (+88%). Decreased pain and frequency of pain were noted (−86% frequency and −50% magnitude, both *p* < 0.05) (Ramachandran et al., 2017, 2018b, 2019, 2020; Dalal et al., 2019). Future work is needed to evaluate the efficacy of such interventions for the prevention of injury and pain syndromes among trainees preparing for a career in aviation.

Other novel interventions have been proposed including trampoline training which is suggested to provide low-intensity, repetitive muscular endurance exercise (Sovelius et al., 2006; Posch et al., 2019). In a study of fighter pilots, trampoline training was found to be equally effective as strength training in reducing in-flight neck strain (Sovelius et al., 2006; Posch et al., 2019). Alternatively, one study in HPJA pilots demonstrated application of traction as a daily home-based intervention using a commercially available unit to mitigate pain (Chumbley et al., 2016).

## Impact Associated With Ejection or Hard-Landing

For HPJA pilots, cervical vertebrae are especially vulnerable to injury during the sudden acceleration of ejection (Kazarian et al., 1980; Jones et al., 2015). Pilots often report previous exposures that may predispose them to chronic pain or degenerative pain including hard landing, autorotation, parachute landings, and exercise injuries (Walters et al., 2013; Jones et al., 2015). For astronauts, the neck is similarly vulnerable with nine instances of trauma to the neck reported in United States astronauts (the eighth leading cause of in-flight musculoskeletal injury to astronauts) (Scheuring et al., 2009). Traumatic injuries are encountered in astronauts both pre-flight and in-flight related to physical training and exercise (Scheuring et al., 2009). Countermeasures to minimize acceleration/deceleration injury in pilots include optimizing the cockpit for impact protection and survivability, airbags, and seat design (Jones et al., 2015).

## Overuse and Ergonomic Injury

Astronauts and aircrew may be at increased risk also because of their increased proclivity toward physical activity and the demands placed on them by rigorous training (Johnston et al., 2010). In astronauts, injuries related to protective equipment were noted in training with the EMU. Astronauts have reported pain and injury to hands, shoulder, feet, arms, legs, neck, trunk, groin, and head (Ramachandran et al., 2018a). These injuries were reported due to overuse in association with suit constraints including its planar hard upper torso, difficulty in donning, glove moisture and fingertip loading, and limited scapulothoracic motion (Ramachandran et al., 2018a). Contributing factors to the development of shoulder injury following EMU training include less recovery time between training sessions, increased frequency of training sessions, and use of the planar version of the hard upper torso (Anderson et al., 2015).

Forced unnatural posture for long periods is another major contributor to musculoskeletal disability in pilots (Pippig, 1994; Phillips, 2011). Many HPJA and RWA pilots report that in order to properly manipulate the controls, they must maintain a forward bend (Kerstman et al., 2012). Some other components of this posture include kyphotic flexion of thoracic and lumbar spine, restricted pelvic motion, extension of the cervical spine, and forward displacement of the center of gravity (Walters et al., 2013). The need to simultaneously manipulate the collective, cyclic, and anti-torque controls requires RWA pilots to maintain their feet on pedals, depriving them of the support of feet stabilized flat on the floor (Walters et al., 2013). Additionally, manipulation of the collective requires leftward isometric flexion of the forearm and shoulder that can lead to muscular fatigue and chronic loading (Walters et al., 2013). Unnatural posture, together with WBV and prolonged use of HSM, contributes to a 67% 12-month prevalence of neck pain, 48% prevalence of low back pain, and 43% prevalence of shoulder pain over the same period in RWA pilots (Posch et al., 2019). Crew have also attributed pain to ineffective seat design including ineffective padding and absence of lumbar support (Phillips, 2011). For astronauts, in-flight neck and shoulder pain during use of the Materials Science Glovebox on Spacelab was attributed to severe neck flexion and a hunched posture required in its operation (Whitmore et al., 1996).

Changes to cockpit equipment have also been considered. For instance, the angle of the seat affects the incidence of pain in pilots of the aircraft (Jones et al., 2000; Johnston et al., 2010). The orientation of the seats in spacecraft may account for the decreased incidence of HNP in astronauts of the Shuttle program compared to long duration ISS missions because the Shuttle astronauts landed vertically (Belavy et al., 2016). Possible interventions include reconfiguration of cockpit seat and flight control geometry (Phillips, 2011).

## Discussion

The unique environmental conditions of flight together with the rigorous training and mission-related tasks conducted by aircrew and astronauts place them at risk for musculoskeletal injuries that have the potential to impact crew readiness and mission success. The astronaut population is small and the relative abundance of HPJA and RWA crew may assist in understanding the musculoskeletal complaints seen in astronauts. One must consider, based on similar type and magnitude of exposures, that if a particular problem manifests in one population, it may be a harbinger of problems to come in the other populations of crew. In this way, collaborative understanding of musculoskeletal issues will help medical providers and researchers to stay ahead of potential issues and develop countermeasures to ensure continued crew health and safety.

The impending launch of commercial spacecraft could pave the way to increased access to space in which case a more diverse population of individuals will be subjected to the musculoskeletal risks of flight. Moreover, interest in long duration missions including those to establish lunar colonies and reach Mars, will result in prolonged exposure to the spaceflight environment beyond that which has been previously studied. These potential developments underscore the importance of understanding the musculoskeletal risks and developing appropriate countermeasures. Even beyond the air and space crew populations, many of these findings and countermeasures may have application to other populations. Neck and back pain affect a significant share of the general non-flying population and pose a similar problem in other occupations including military parachutists, infantry soldiers, and law enforcement largely as a result of eccentrically loaded HSM (Estep et al., 2019), as well as athletes especially those in contact sports.

As a result of the occupational hazards of the flight, crew are exposed to numerous risks that have the potential to impact crew health and readiness. Every effort must be made to address these risks and implement effective countermeasures. The risks and exposures are varied and there is evidence of both acute and chronic injuries in crew. The resulting distribution of pain across age groups is bimodal, with the largest share of those affected being either early career aviators being introduced to flight or those accumulating more hours late in their career (Jones et al., 2000). In the first group, the pain could be best understood because of adaption to new forces on an unconditioned body. In more experienced crew, the pain is understood as a degenerative change or inability of an aging body to perform under repeated stress, a concept that is supported by MRI evidence of increased degenerative cervical spine changes in the IVD of pilots (Johnston et al., 2010).

The goal of countermeasures is to prevent irreversible injury but, in some cases, when this is not possible, countermeasures must be employed to mitigate existing injury or rehabilitate patients. Some examples of countermeasures for mitigation of existing injury include pharmacological interventions such as anti-inflammatory medications, massage, acupuncture, heating pad, traction, physiotherapy, and chiropractic or osteopathic manipulation (Pippig, 1994; Kikukawa et al., 1995; Kerstman et al., 2012; Thoolen and van den Oord, 2015). However, some of these interventions have been associated with complications including vertebral artery dissection and cervical spine myelopathy (Connolly, 2014).

Numerous approaches have been taken to develop countermeasures including those focused on aircraft design, crew strength and behavior, and protective equipment worn by crew. Each approach has its unique advantages and disadvantages. Aircraft design interventions, for example, are likely to require the most upfront cost but impose the least time and effort burden on crew. While exposures are similar between crew, interventions may need to be individually tailored to the airframe. For instance, seat interventions in HPJA require titration of seatback angle to optimize G-tolerance with lowered incidence of neck pain. In RWA, seat interventions require focus not only on pilot postural support but also on vibration reduction and crash-landing protection. Vibration reduction can be tackled not only through seating interventions, but also through improved engine and airframe design.

Effective countermeasure development must be crew-friendly and take into account barriers to implementation. Time limitations imposed by busy flying schedules result in poor adherence to exercise countermeasures (DeHart, 1985). Other barriers to effective exercise countermeasures include insufficient availability of experienced training staff or weight-training facilities (Kikukawa et al., 1995). The time constraints, together with frequent deployment and relocation of military personnel necessitates that interventions be accessible, limited in time commitment, and portable. Furthermore, access to necessary equipment has proven to be a potential barrier to countermeasure use. For example, one group of RWA pilots reported that counterweights were difficult to acquire through their supply chain (Phillips, 2011). Poor adherence has been implicated in failure to achieve pain relief. In one study, only one-third of subjects engaged in the prescribed exercises at least once per week (Murray et al., 2017). Other studies have reported adherence to exercise regimens between 52 and 77% (Ang et al., 2009; Salmon et al., 2013). Countermeasures in spaceflight should also take into account the constraints on weight and volume imposed by the mass to orbit cost of rocket launches, which favors the use of more simple and low-weight devices for astronauts.

It is important that countermeasures are made available to all crew who may be at times more vulnerable to injury than the pilots (Kikukawa et al., 1995). Backseat crew in HPJA such as navigators, radar intercept and weapons officers are unable to brace themselves and are exposed to unexpected abrupt movements (Kikukawa et al., 1995; Lounsbury et al., 2002). Interventions should also account for individual differences in crew and understand that different pathologies must be intervened upon differently. For example, the etiology of spine pain in younger pilots is often due to a bony spondylosis while in older aviators it is most commonly due to inability of a degenerative IVD to withstand compressive force leading to nerve root compression (Connolly, 2014). While it is sensible to target problem areas in crew with active injuries, preventive interventions must be comprehensive to protect against a spectrum of injuries. For example, ongoing efforts are seeking to identify risks to the whole spine (both neck and back) in U.S. Naval aircrew (Le, 2019).

It is imperative to acknowledge the limitations of exercise countermeasures alone, because some individuals have certain congenital or pre-disposing conditions that either make the countermeasures ineffective or unacceptably elevate the risks of flight. Because of the considerable investment of time and resources in the training of air crew and astronauts, imaging studies may be conducted to identify individuals with certain pathology. Plain film x-ray, for example, may be used to identify spine conditions such as spondylosis, spondylolisthesis, and spina bifida occulta in prospective aviators (Kikukawa et al., 1995). Furthermore, the countermeasures must because designed with safeguards so as not do more harm than good. Exercise interventions in spaceflight including the interim and advanced resistive exercise device, and the TVIS have been associated with musculoskeletal injuries in astronaut users (Scheuring, 2016). Accordingly, active monitoring of countermeasure implementation is warranted to ensure that it is achieving intended effects and potential shortcomings can be addressed.

The results of studies on cervical resistive exercise suggest that a self-directed portable exercise device is easy for mobile aircrew to employ and can increase neck muscle endurance and cervical spine range of motion and reduce neck pain frequency in pilots. These results and future studies should inform the resources made available and timing of intervention for pilots to ensure crew health and safety, prevent medical disqualification, and enable mission-readiness. This type of intervention could also potentially prove to be applicable for astronauts and other personnel exposed to similar risks of flight. Further effort is needed to extend enrollment to demonstrate reproducible results in a more varied cohort of patients in both training and operational phases.

The wide variety of approaches available to counteract the effects of flight on crew together with the preliminary success of portable, self-directed exercise intervention provide promising relief to personnel putting themselves at risk to carry out their missions in an aerospace environment. Further investigations are required to fully elucidate the scope, risk factors, mechanisms, and anatomical structures involved in musculoskeletal pathology in aviation and spaceflight environments to continue to refine the available countermeasures.

## Data Availability Statement

All relevant data is contained within the article: the original contributions presented in the study are included in the article, further inquiries can be directed to the corresponding author.

## Ethics Statement

Written informed consent was obtained from the individuals for the publication of any potentially identifiable images or data included in this article.

## Author Contributions

DO’C conducted the literature review. DO’C, SD, and VR prepared the manuscript. JJ, BS, and BSS developed the concept of the project and oversaw its execution and edited and augmented the manuscript. All authors contributed to the article and approved the submitted version.

## Conflict of Interest

The authors declare that the research was conducted in the absence of any commercial or financial relationships that could be construed as a potential conflict of interest.
